# Acutely enhancing affective state and social connection following an online dance intervention during the COVID-19 social isolation crisis

**DOI:** 10.1186/s40359-022-01034-w

**Published:** 2023-01-16

**Authors:** Ashlee Humphries, Noor Tasnim, Rachel Rugh, Morgan Patrick, Julia C. Basso

**Affiliations:** 1 Department of Human Nutrition, Foods, and Exercise, Virginia Tech, Integrated Life Sciences Building, 1981 Kraft Drive, Blacksburg, VA 24016 USA; 2 Graduate Program in Translational Biology, Medicine, and Health, Virginia Tech, Blacksburg, VA USA; 3 School of Performing Arts, Virginia Tech, Blacksburg, VA USA; 4 School of Neuroscience, Virginia Tech, Blacksburg, VA USA; 5 Center for Research On Health Behaviors, Fralin Biomedical Research Institute at VTC, Roanoke, VA USA

**Keywords:** Affect, Mood, Movement, Exercise, Physical activity

## Abstract

The COVID-19 pandemic has forced many throughout the world to isolate themselves from their respective communities to stop the spread of disease. Although this form of distancing can prevent the contraction of a virus, it results in social isolation and physical inactivity. Consequently, our communities have become heavily reliant on digital solutions to foster social connection and increase physical activity when forced to isolate. Dance is a multidimensional form of physical activity that includes sensory, motor, cognitive, rhythmic, creative, and social elements. Long-term, interventional studies in dance have shown positive effects on both mental and social health; however, little has been done to examine the acute effects and no studies to date have explored the relationship between the affective state and social outcomes of dance. We examined the hypothesis that online dance is associated with improvements in affective state and social connection during a time of social isolation, namely, the COVID-19 crisis. Healthy adults (age ≥ 18; n = 47) engaged in a single session of 60 min of self-selected online dance, completing a series of validated self-reported questionnaires before and after class. We found that online dance was associated with improvements in affective state as measured by increased positive affect and self-esteem and decreased negative affect and depressive symptoms. Additionally, online dance was associated with improvements in social and community connectedness. Further, we found that those who experienced the largest increases in self-esteem and decreases in negative affect demonstrated the largest gains in social connectivity. Although in-person dance classes may be optimal for formalized dance training, online dance instruction offers an accessible platform that can provide mental and social health benefits during the COVID-19 social isolation crisis. We conclude that through online dance, individuals can experience a connection between the body, mind, and community.

## Introduction

The COVID-19 pandemic has forced populations throughout the world to distance themselves from their respective communities. Although this tactic effectively slows down the spread of the virus, it results in social isolation and physical inactivity. Consequently, the COVID-19 pandemic increased rates of social isolation from as low as 10% across the USA, Europe, and China to upwards of 24%, with millennials experiencing loneliness more than any other age group [1, 2]. Social isolation can not only exacerbate current symptoms of mental and physical health disorders, but also increase the risk of developing mental health issues in otherwise healthy adults. Recent studies detail the mental health impacts of COVID-19, including increased symptoms of anxiety, depression, stress, avoidance behaviors, insomnia, anger, and fear [3, 4]. These alarming symptoms call for a need to address an increasing mental health crisis along with the physical health crisis we face. Our communities have become heavily reliant on digital solutions to foster social connection and increase physical activity when forced to isolate. Video calls and online exercise routines have notably alleviated the detrimental effects of being stuck at home. Recent literature shows that in the middle of a pandemic, exercise has been particularly beneficial in boosting mood, especially in combatting the effects of depression, stress, and exhaustion, all of which are often experienced by people in quarantine [5].

The positive effects of exercise on mental health are well established, both in terms of acute and long-term effects [6, 7]. These effects include but are not limited to, mood enhancement, anxiety prevention, and sleep regulation [8], with a significant negative relationship existing between physical activity level and depression and anxiety disorders [9–11]. Dance differs from other physical activities in that dance is a multimodal artistic practice that incorporates aspects of sensory, motor, cognitive, social, emotional, rhythmic, and creative processes [12]. In this way, we can think of dance as an enriched form of physical activity. Animal studies have shown that the combination of physical exercise with sensory enrichment has the most prominent effect on the survival of new neurons and their integration into brain circuits [13, 14]. Though exercise is one of the best ways to promote neurogenesis (i.e., the birth of new neurons), other cognitive enrichment experiences are needed for those new neurons to become an integral part of the brain and contribute to its functional activity. In fact, studies suggest that dance may be superior to aerobic exercise as a way to increase brain plasticity and possibly prevent or delay impairments in mood or cognition in both healthy, aging, and other clinical populations [15, 16].

Previous research has shown that dance is beneficial for mental health issues, including depression, anxiety, schizophrenia, and disordered eating [17–19]. Qualitative studies have shown that dance reduces self-reported symptoms of depression, with participants providing four primary reasons for the beneficial effects, including dancing for their own health, social acceptance, connection with others, and not wanting to stop due to unexpected benefits [20]. Additionally, dance has been shown to reduce psychological distress and improve stress management, with effects lasting up to 6 months after the intervention [21]. Importantly, dance has also been shown to improve mental well-being among non-clinical populations, especially amongst children and the elderly [17–19]. In the realm of improving social connection, dance has also shown favorable outcomes. Murrock and Graor [20] studied disadvantaged adults who experienced depression from social isolation and their response to a 12-week dance intervention. They found that participants developed feelings of social acceptance, connection with others, and an adoption of a group identity. Further, Bognar et al. [22] found that patients with Parkinson’s disease developed a sense of social connectedness through dance, improving an outlook on life and decreasing feelings of negativity toward disease diagnosis. These studies focused on long-term interventions of at least 6 weeks in duration; however, a significant gap in the literature is the focus on the acute effects of dance on psychological health, especially during the COVID-19 isolation crisis. Although previous books and articles in dance studies and anthropology have studied the connection between dance, community, and mental health, it is worth conducting further study in the context of a global pandemic and forced isolation.

Due to stay-at-home orders, social media, video conferencing, and other forms of digital communication have seen an increased use, not only for personal reasons but for conducting necessary work and school functions [23]. Dance classes have made a similar shift; since March 2020, many studios, companies, and teams have transitioned to online collaboration or have canceled in-person movement classes completely. This presents a unique challenge to dance teachers as they learn how to use a two-dimensional format of education for a skill that is so three-dimensional in nature. Gingrasso [24] identifies several obstacles in virtual dance instruction, such as an inability to give tactile cues or not being able to see the whole body in movement due to screen restrictions. However, while some may argue that online learning inhibits social connection, Smith-Merry et al. [25] identifies online forums as a useful tool in maintaining social connectivity during times when a face-to-face connection is limited. We hypothesize that online dance instruction may be a way to combat social isolation and mental health issues resulting from the pandemic.

For individuals experiencing social isolation during the pandemic, it is of importance to find easy-access interventions that improve mental health and more generally affective state. We hypothesized that a single 60-min session of online dance would be significantly associated with decreases in negative affective states and increases in positive affective states. It is important to note that this single session is not Dance Movement Therapy (DMT) and that this study examined the effects of dance class, in general. We also hypothesized that dance would be significantly associated with an increased level of social connectivity and that the affective state changes would predict these social outcomes. Collectively, the present study assessed the effects of an online dance class on affective state and social connectivity in healthy adults during the COVID-19 pandemic.

## Methods

This acute experimental study consisted of a repeated-measures design; participants took a self-report, questionnaire-based assessment both before and after an online dance class. Participants were openly recruited via online communication through social media, advertisements on Dancing Alone Together (dancingalonetogether.org), networking with Blacksburg Dance Theater (blacksburgdancetheater.com), and Virginia Tech’s Listserv resources. Interest emails were directed to the Embodied Brain Lab (embodiedbrainlab.com), where participants signed up for a class using their email addresses. Once participants signed up for the class using Sign Up Genius (signupgenius.com), they received an email with a link to the pre-participation Qualtrics survey. Potential participants were eligible to complete both pre- and post-intervention surveys if they were age 18 or older, living within the United States, and English speaking (since the dance classes were taught in English). Participants also needed to pass the Physical Activity Readiness Questionnaire (PAR-Q) [26] before participating to indicate capability of engaging in physical activity. Additional exclusionary criteria included not ambulatory and intellectual impairment that would impact psychological assessment or intervention participation, as our aim was to study primarily healthy populations. Participants were screened for former or current psychiatric diagnoses and medication intake, but these did not serve as exclusionary criteria; rather, these pieces of information provided researchers with a broader picture of the mental and physical health of the individual. All methods were approved by the Virginia Tech Committee on Activities Involving Human Subjects and were performed in accordance with all relevant guidelines and regulations.

During the pre-intervention survey, participants signed an electronic informed consent, then answered a series of questions to assess demographic information, including age, gender, race, ethnicity, employment status, education, income, living environment, height, weight, and body mass index (BMI).

### Affective state measures

Our primary study endpoints included affective state measures, which were assessed using the following scales: (1) self-esteem as assessed by the Rosenberg Self-Esteem Scale [27]; (2) positive and negative affect as assessed by the Positive and Negative Affect Schedule-Short Form [28]; (3) anxiety as assessed by the Beck Anxiety Inventory [29]; and (4) depressive symptoms as assessed by the Beck Depression Inventory [30].

Rosenberg Self-Esteem Scale [27]: The Rosenberg Self-Esteem Scale (RSES) is the most widely used self-esteem measure; it consists of a 10-item scale that measures positive and negative feelings towards oneself using a 4-point Likert scale. Total scores range from 0 to 30, with a score lower than 15 suggesting low self-esteem (Cronbach’s alpha = 0.72–0.87).

Positive and Negative Affect Schedule-Short Form [28]: The Positive and Negative Affect Schedule-Short Form (PANAS-SF) is a reliable and validated questionnaire consisting of two 10-item scales that assess mood using a 5-point Likert scale. These two scales assess both positive (PA) and negative affect (NA); the sum of the items for PA and NA are scored separately and each range from 10 to 50 (Cronbach’s alpha = 0.84–0.90).

Beck Anxiety Inventory [29]: The Beck Anxiety Inventory (BAI) is a valid and reliable tool consisting of a 21-item questionnaire, where items are scored using a 4-point Likert scale. The sum of the items yields a total anxiety score ranging from 0 to 63 (Cronbach’s alpha = 0.83–0.92).

Beck Depression Inventory [30]: The Beck Depression Inventory (BDI) is a consistent and reliable tool that utilizes a 21-item self-reported inventory to measure the severity of depressive symptoms (Cronbach’s alpha = 0.92).

### Social connection measures

Social measures, another primary endpoint, included the following: (1) social connectedness as assessed by the Social Connectedness Scale [17]; (2) loneliness as assessed by the UCLA Loneliness Scale [31]; and (3) community inclusion as assessed by the Inclusion of Community in Self Scale [32].

Social Connectedness Scale [17]: The Social Connectedness Scale (SCS) is a highly reliable and valid tool used to assess the degree of an individual’s connection to others within their social environment. It utilizes a 20-item questionnaire that is scored using a 6-point Likert scale (Cronbach’s alpha = 0.94).

UCLA Loneliness Scale [31]: The UCLA Loneliness Scale is a validated tool that measures a person’s subjective feelings of loneliness and social isolation. It is a 20-item questionnaire that allows participants to self-rate each item as “often,” “sometimes,” “rarely,” or “never” (Cronbach’s alpha = 0.96).

Inclusion of Community in Self Scale [32]: The Inclusion of Community in Self Scale (ICS) is a reliable and valid single-item measure of community connectedness. Participants self-select one of seven pictorial models that best identify their feelings towards their relationship to their community, represented by circles that converge to varying degrees.

Subjective Exercise Experiences Scale [33]: The Subjective Exercise Experiences Scale (SEES) assesses three categories of subjective response to exercise: positive well-being, psychological distress, and fatigue. It is a questionnaire that is highly consistent across a variety of populations and consists of a 12-item scale, with each item rated using a 7-point Likert scale (Cronbach’s alpha = 0.85–0.88).

Study personnel reviewed the pre-intervention assessment to ensure that each participant was eligible for the study. If the participant was deemed ineligible, they were sent an email informing them of their inability to be included in the study. Eligible participants were sent a standard email confirming their participation with the date and time of their scheduled dance class. Twenty-four hours prior to the class, participants were sent a reminder and a Zoom link to the class. At their scheduled time, participants logged into Zoom and engaged in a 60-min online dance class. Participants were asked to activate their camera (to ensure similar levels of engagement with the instructor and other participants) but had the option to mute their microphones. Additionally, participants were reminded to clear an approximately 8- by 10-foot space to minimize any risks associated with dancing in their home. Furthermore, choreography was taught, and group performance/observation was included at the end of class. These classes were taught by Blacksburg Dance Theater faculty and included three genres of dance: modern, ballet, and jazz. All instructors were given a brief overview of the general study protocol. All classes included 1 main instructor. The instructor could see everyone on the screen. Two out of the five instructors had college or advanced degrees in dance (i.e., MFA), while the other three had varying years of dance experience (10–20 years of teaching experience). Participants were able to self-select their preferred dance genre prior to the class. All classes were taught at a beginner level, but movement options were offered for each dance exercise to accommodate dancers of all skill levels. Participants received the dance class at no cost. Participants ranged from beginner to professional dancers [34].

Thirty minutes into the dance class, the participants received an emailed link to a post-intervention Qualtrics survey; this was to encourage the greatest adherence in finishing both surveys by having the email already waiting in their inbox when class ended. At the end of class, dance instructors reminded the participants to complete the survey within two hours after the Zoom meeting ended. This interval was selected because the greatest number of acute physiological effects of exercise exist within 120 min after cessation of activity [6]. All primary endpoint measures were re-assessed in the post-intervention survey as part of the repeated-measures study design. The SEES was only given at the post-intervention survey as a secondary endpoint measure.

### Data analysis

To determine the sample size to sufficiently power our study, an a-priori power analysis was conducted using the UCSF Clinical and Translational Science Institute correlation sample size calculator (https://sample-size.net/). The power analysis was based on a correlation with an alpha (ɑ) of 0.05, a beta (β) of 0.20, and a correlation coefficient (r) of 0.4, with results indicating a sample size of n = 47.

**Fig. 1:**
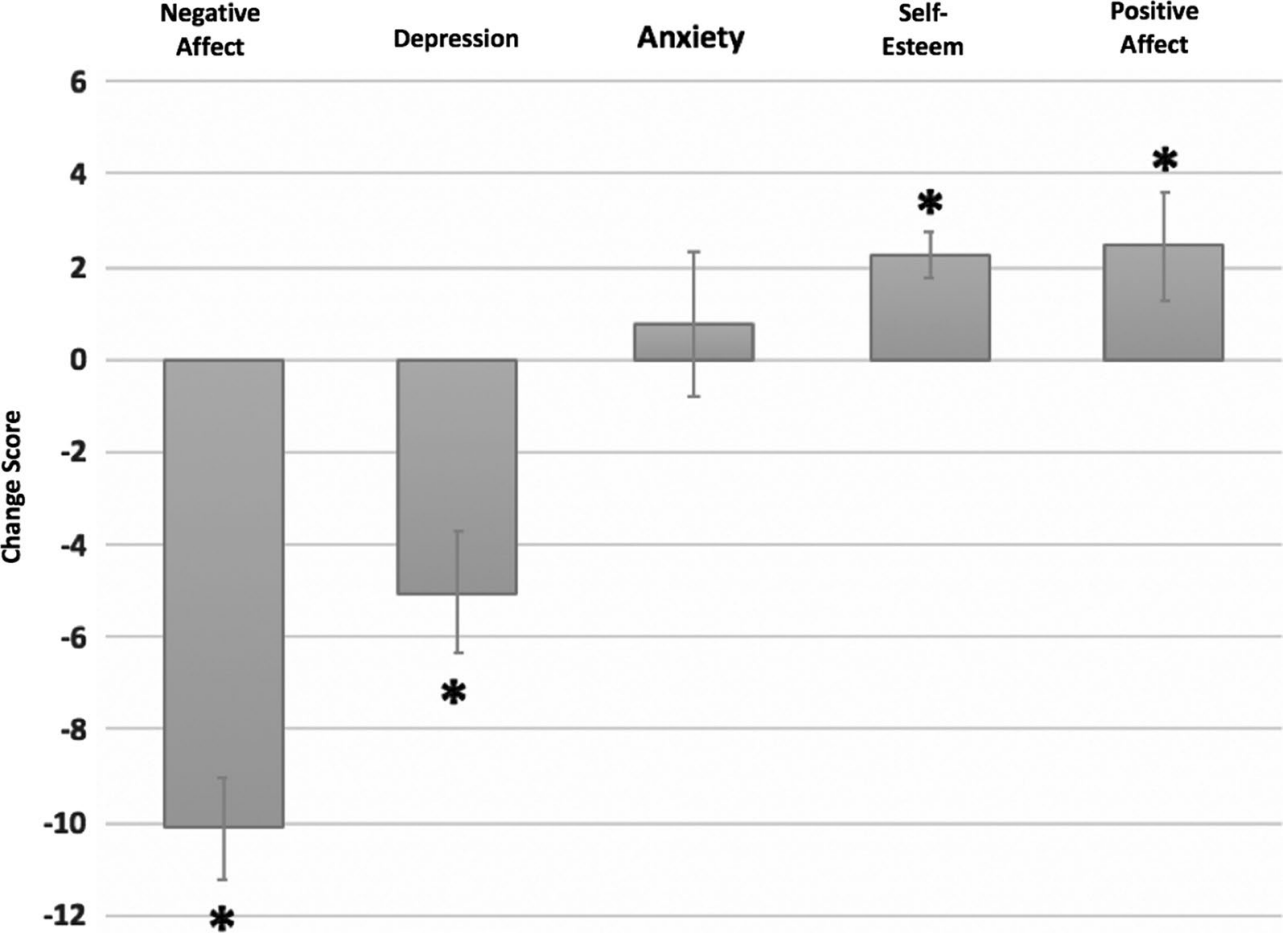
A single session of online dance was significantly associated with decreases in negative affect and depressive symptoms and increases in self-esteem and positive affect. “Change score” on the y axis represents pre-intervention scores subtracted from post-intervention scores. *Statistically significant at p < 0.05

**Fig. 2:**
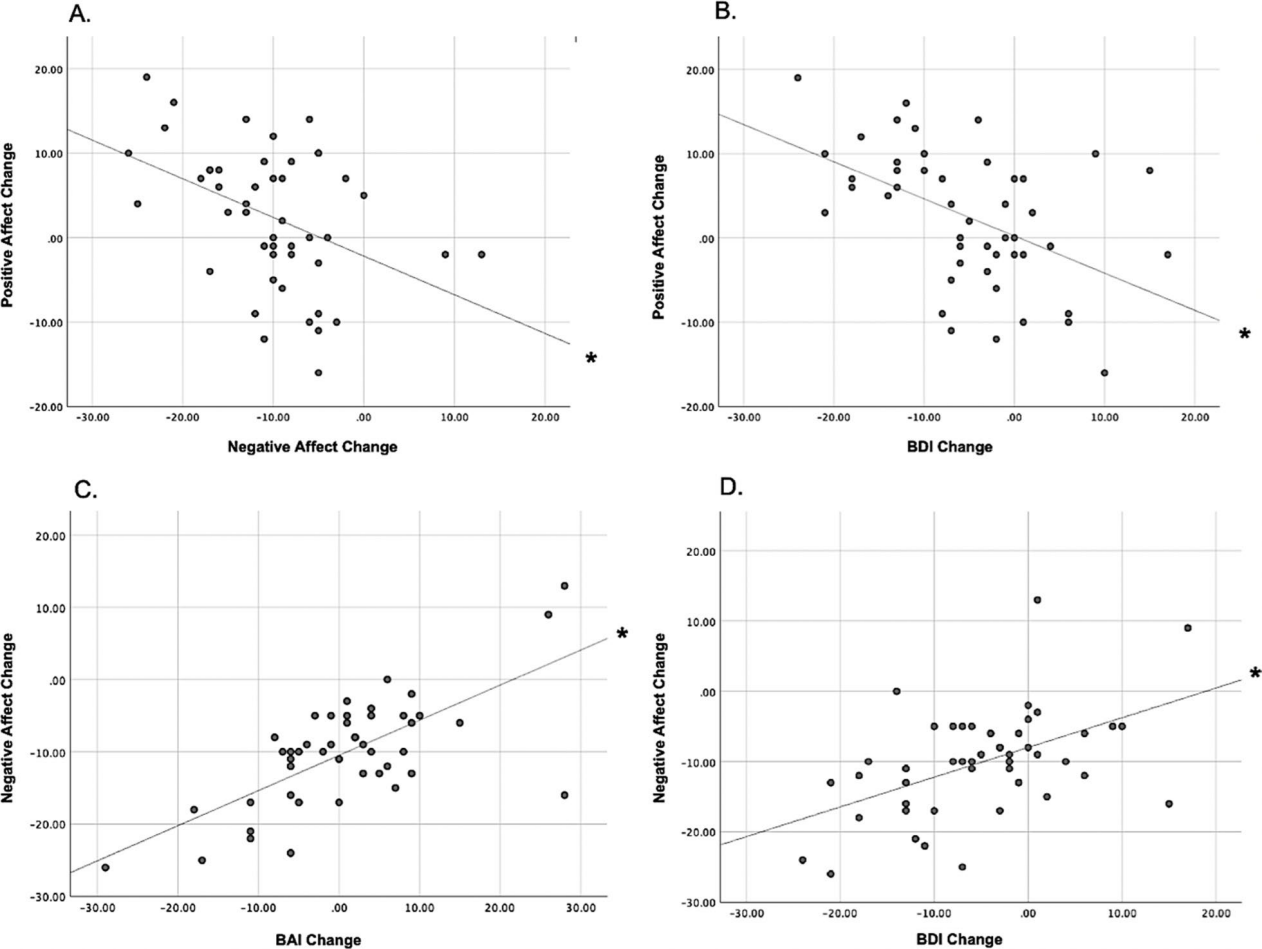
Relationship between primary affective state outcomes. **A**, **B** The dance-induced change in positive affect showed a significant negative association to the change in negative affect and depressive symptoms. **C**, **D** The dance-induced change in negative affect showed a significant positive association to the change in anxiety and depressive symptoms. Scores on the y axis represent pre-intervention scores subtracted from post-intervention scores. *Statistically significant at p < 0.05

**Fig. 3:**
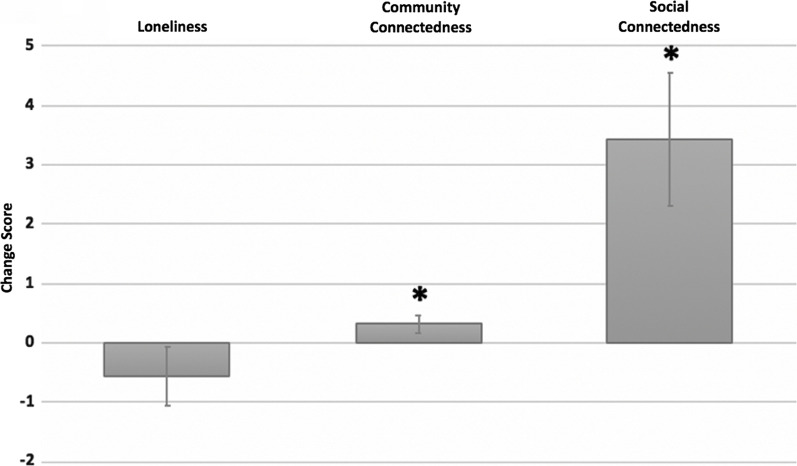
A single session of online dance was significantly associated with enhancements in community and social connectedness. “Change score” on the y axis represents pre-intervention scores subtracted from post-intervention scores. *Statistically significant at p < 0.05

**Fig. 4:**
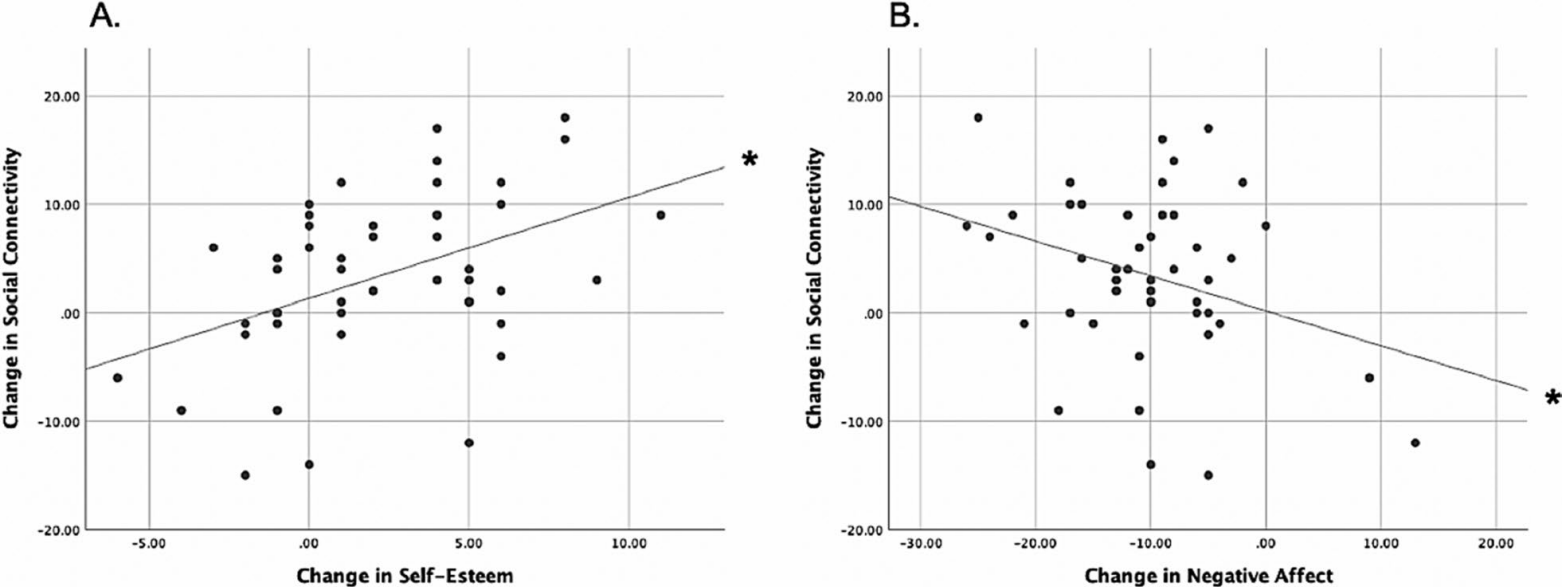
**A** Dance-induced increases in self-esteem were significantly associated with enhancements in social connection. **B** Dance-induced decreases in negative affect were significantly associated with enhancements in social connection. Scores on the x and y axes represent pre-intervention scores subtracted from post-intervention scores. *Statistically significant at p < 0.05

All data were analyzed using SPSS Statistics 26.0 [35]. Change scores were calculated by subtracting the pre-intervention from the post-intervention measures. These scores were used to determine the impact of the dance intervention and are displayed in Figs. 1, 2, 3 and 4. Two-sided paired-samples t-tests were used to determine whether there was a statistically significant difference between the pre-intervention and post-intervention outcomes for all affective state and social connectivity measures. Further, we used Pearson’s product-moment correlations to examine the relationship between changes in affective state measures as well as the subjective exercise experience. Finally, we used linear regression models to analyze the predictive validity of dance-induced changes in affective state on changes in social connection. For all analyses, statistical significance was assessed utilizing an alpha value of 0.05.

## Results

### Demographics

We collected data from n = 59 participants; however, the final number of participants who completed the dance class and all research procedures was n = 47. The age of participants ranged from 18 to 70, with a mean age of 32.47 years (± 2.32 SEM). Of these participants, 68% had an annual household income in the middle- or high-income categories, 92% had a college or advanced degree, and 38% worked full time (Table 1). Our study contained more female (91%) than male participants and were primarily of Caucasian descent (77%) and non-Hispanic (98%). Urban and rural communities were nearly equally represented at 49% and 51%, respectively. Further, 55% of participants were in the BMI range for normal weight (BMI of 18.5–24.9), compared to the national obesity prevalence of 42.4% in adults [36].

**Table 1:** Demographic information regarding our participant population

A. Frequency table		B. Contigency table	
Mean age = 32.47		n = 47	
Household income		Household income	
% Low income	32%	Low income	15
% Middle income	47%	Middle income	22
% High income	21%	High income	10
Education		Education	
% High school/GED or lower	4%	High school/GED or lower	2
% Some college	4%	Some college	2
% College degree	43%	College degree	20
% Advanced degree	49%	Advanced degree	23
Employment Status		Employment Status	
% Working full time	38%	Working full time	18
% Working part time	38%	Working part time	18
% Not working	23%	Not working	11
Sex		Sex	
% Female	91%	Female	43
% Male	9%	Male	4
Race		Race	
% White/Caucasian	77%	White/Caucasian	36
% Black/African American	6%	Black/African American	3
% Asian	13%	Asian	6
% Other	4%	Other	2
Ethnicity		Ethnicity	
% Hispanic	2%	Hispanic	1
% Non-hispanic	98%	Non-hispanic	46
Urban versus rural community		Urban versus rural community	
% Urban	49%	Urban	23
% Rural	51%	Rural	24
BMI		BMI	
% Underweight (< 18.5)	2%	Underweight (< 18.5)	1
% Normal Weight (18.5–24.9)	55%	Normal Weight (18.5–24.9)	26
% Overweight (25–29.9)	23%	Overweight (25–29.9)	11
% Obese (> 30)	19%	Obese (> 30)	9

### Affective state outcomes

#### Acute effects of dance on affective state

After the dance class and compared to baseline measures, participants experienced increases in positive affect (t(46) = − 2.067, p = 0.044) and self-esteem (t(46) = − 4.264, p < 0.0001) as well as decreases in negative affect (t(46) = 9.166, p < 0.001) and depressive symptoms (t(46) = 3.808, < 0.001) (Fig. 1). Anxiety measures showed no statistically significant differences (p > 0.05).

We further assessed the relationships between our five affective state measures using Pearson’s product-moment correlations (Fig. 2; Table 2). There was a statistically significant negative correlation between positive affect and negative affect change scores (Fig. 2A; r = − 0.427, p = 0.003), as well as a negative correlation between positive affect and BDI change scores (Fig. 2B; r = − 0.493, p < 0.0001). Change in positive affect additionally yielded a significant positive correlation to the change in self-esteem (r =  0.317, p = 0.030). Further, the change in negative affect was positively correlated with BAI (Fig. 2C; r = 0.691, p < 0.0001) and BDI change scores (Fig. 2D; r = 0.507, p < 0.0001). The correlation between BAI and BDI change scores was also significant (r = 0.629, p < 0.0001).

**Table 2:** Pearson’s correlations table for dance-induced affective state and social connection change scores

	1	2	3	4	5	6	7	8
1. Positive affect								
2. Negative affect	− 0.427**							
3. Anxiety	− 0.187	0.691**						
4. Depression	− 0.493**	0.507**	0.629**					
5. Self-esteem	0.317*	− 0.235	− 0.105	− 0.262				
6. Loneliness	0.051	− 0.168	− 0.063	− 0.004	− 0.009			
7. Community inclusion	0.196	− 0.295*	− 0.068	− 0.139	0.420**	0.245		
8. Social connectedness	0.094	− 0.317*	− 0.266	− 0.112	0.441**	0.164	0.328*	

*p < 0.05; **p < 0.01

#### The psychological response to exercise is associated with changes in affective state

SEES positive well-being was positively associated with the change in positive affect experienced after dance (r = 0.356, p = 0.014). SEES positive well-being was also negatively associated with the change in anxiety experienced after dancing (r = − 0.320, p = 0.028). Psychological distress was positively associated with the change in both anxiety (r = 0.567, p < 0.0001) and depression (r = 0.328, p = 0.025). Fatigue levels also demonstrated positive correlations with the change in psychological distress (r = 0.622, p < 0.0001) and anxiety (r = 0.422, p = 0.003).

### Social connection outcomes

#### Acute effects of dance on social connectivity

Online dance significantly enhanced social connection (t(46) = − 3.069, p = 0.004) and community connection (t(46) = − 2.183, p = 0.034) (Fig. 3). Positive correlations were also seen between social and community connectedness (r = 0.328, p = 0.025). There were no statistically significant effects associated with the UCLA Loneliness Scale (p > 0.05).

#### The affective state response predicts social connectedness

Those who experienced the largest decreases in negative affect also demonstrated the largest gains in social (r = − 0.317, p = 0.030) and community connectivity (r = − 0.295, p = 0.044). Further, we found that participants with the greatest increases in self-esteem experienced the largest gains in social (r =  0.441, p = 0.002) and community (r = 0.420, p = 0.003) connectedness.

A linear regression model established that dance-induced self-esteem increases statistically significantly predicted enhanced social connection (Fig. 4A; F(1, 45) = 10.863, p = 0.002), with improvements in self-esteem accounting for 19.4% of the explained variability in social connectivity increases with adjusted R^2^ = 17.7%. To assess linearity, a scatterplot of social connectivity against self-esteem change with a superimposed regression line was plotted. Visual inspection of these two plots indicated a positive linear relationship between the variables. An identical linear regression model established that dance-induced decreases in negative affect predicted enhanced social connection (Fig. 4B; F(1, 45) = 5.044, p = 0.030). Negative affect accounted for 10.1% of social connection variability with adjusted R^2^ = 8.1%. A visual inspection of the plotted regression line indicated a negative variable relationship.

## Discussion

The current study examined the effects of an acute session of online dance on affective state and social connection during the COVID-19 crisis. We found that online dance was associated with acute decreases in negative affect and depressive symptoms and enhancements in positive affect and self-esteem. Additionally, we found that the subjective experience of dance was significantly associated with the change in affective state. Further, we found that one session of online dance was associated with acute enhancements in social and community connectedness. Importantly, the change in affective state significantly predicted the change in social connection. This suggests that dance, even via an online platform, can be used to improve mental and social health, suggesting a body-mind-community connection. A body-mind-community connection is a linkage between physical, mental, and social health. These findings have important implications for adult populations dealing with social isolation and resulting mental health issues during times of a pandemic or otherwise. It is important to note that our sample consisted of healthy adult dancers without mental health issues. Our findings imply that online dance may have acute mental health benefits for this population; however, we have yet to extrapolate these findings to a clinical population. We do, however, suggest that online platforms can be used effectively to disseminate dance to diverse populations. Online instruction makes dance more accessible, and its digital format can help to reach marginalized populations that would not be able to attend dance lessons otherwise. Moreover, this wide reach can be economically advantageous by lessening the cost of travel. Health care personnel and frontline workers, in particular, could benefit from this intervention as these individuals need to work long hours in the hospital; online access to dance may be critical when they are unable to go home or attend in-person classes.

### Dance is associated with acute enhancements in affective state

Regarding affective state, our data shows that a dance intervention is associated with increased positive affect and self-esteem while minimizing negative affect and depressive symptoms. Further, decreases in negative affect and depressive symptoms were significantly associated with both decreases in anxiety and increases in positive affect. While several researchers have examined the effects of dance on depression and anxiety in clinical populations, fewer studies have specifically analyzed positive and negative affect. Current research demonstrates that DMT focused on elements associated with happiness can significantly enhance feelings such as empowerment, pride, and determination, which are part of positive affective states [37]. Although our current study did not examine the effect of DMT, specifically, our observations on the effects of dance, in general, match those from DMT. More closely related to our study, Koch et al. [38] investigated the impacts of a single dance movement session on depression and positive affect in 31 psychiatric patients diagnosed with depression. Comparing dance, listening to music, and riding a stationary ergometer, they found that the dance group profited most in terms of decreased depression and more vitality. Additionally, a recent, large randomized controlled trial (RCT) demonstrated that DMT reduced negative affect, depression, and loneliness in older adults with mild dementia whereas exercise alone did not [39]. These findings are analogous to our study; a similar effect was seen after a single dance session, suggesting that a dance class is at least as effective, if not more effective, in improving affective states when compared to other forms of exercise. Our study was not designed as an RCT and did not explicitly compare dance to other forms of exercise but adds to the knowledge of the effects of a single session of dance for healthy adults.

### Dance is associated with acute enhancements in social connectivity

Current evolutionary theories posit that dance has evolved as a form of imitation for the purposes of social communication, connection, and learning [40]. The current study demonstrated that online dance was associated with significant enhancements in social connection and community connection with positive correlations seen between social and community connectedness. This finding coincides with the social alignment theory, where motor, cognitive, and emotional synchrony happens as humans build relationships and activate areas of the brain associated with the action observation network (e.g., premotor cortex, superior temporal sulcus, superior parietal lobe) [12, 41, 42]. As individuals enter a dance practice, they use motor and cognitive areas of the brain to process and execute choreography, but they also exhibit emotional expression through their movements. These three elements then contribute to synchronization and harmony of movement with other dancers, increasing feelings of social connectivity [43]. In our study, as participants danced, social alignment and connection took place without physically being in the same room as the other participants. Previous research has shown that implementing social inclusion strategies through an online-based forum is strongly suggested in the mitigation of negative effects from confinement [44]. Our research is notable in the fact that it is the first to study the link between dance and social connection in healthy populations.

### Subjective exercise experience is associated with the acute affective state response of dance

The subjective exercise experience also influenced affective state in that it was a significant predictor of affective state changes. This finding is similar to other work showing that individuals who participated in movement with other dancers showed an increase in subjective enjoyment of the dance experience [45]. In our study, we saw that the positive well-being experienced from dance enhanced positive affect and decreased feelings of anxiety. This finding is supported by the research of Campion and Levita [46] who compared the effect of dance on affect and cognition to music or exercise in a young, non-clinical population. Their research demonstrated that both dancing and passively listening to music enhanced positive affect, decreased negative affect, and reduced feelings of fatigue. Our results also demonstrated that those who experienced psychological distress and fatigue in response to acute dance had amplified anxiety and depressive symptoms. It is possible that this finding could be related to perceived class difficulty level and corresponding increases in anxiety.

### Dance and the body-mind-community connection

Importantly, we found that the acute effects of dance on affective state significantly predicted the change in social connection. Specifically, those individuals who demonstrated the largest gains in self-esteem and decreases in negative affect showed the largest enhancements in social connectivity. This is the first time that this relationship has been investigated in the context of an acute dance protocol.

Other work has shown that mental health and social connectivity are inextricably linked. Individuals with low levels of social connectedness show impaired mental and physical health, including increased levels of depression and shorter life expectancies than those with strong social bonds [47, 48]. In fact, in the realms of public health and epidemiology, it is well accepted that social connection acts as a protective mechanism against mental illness [49, 50]. Conversely, mental health impacts one’s ability to engage in social interactions, and there is often a lack of social connection in individuals with depression, anxiety, or substance use disorders due to dysregulated interpersonal processes [51, 52].

In related work, one study found that a 4-week physical-activity based youth development program for low-income youth improved social and physical competence as well as physical and global self-worth [53]. Further, they found that the changes in self-competence predicted the changes in mental health measures including self-worth and hope. Additionally, a 3-month Gerofit exercise program in older Veterans significantly improved posttraumatic stress disorder symptoms, and this improvement was significantly associated with the level of social connectedness [54].

Our work shows that dance, even in an online platform, is associated with enhancements in both affective state and social connection and that these effects are integrally linked. We hypothesize that our video conference software, which allowed participants to see each other during all dance instruction, significantly contributed to this effect. Future research should investigate which aspects of the online platform (e.g., camera on versus off; gallery view versus speaker view) support enhancements in mental and social health. Additionally, online physical activity programs that have a social component, such as the one in the current study, have been shown to enhance engagement in physical activity. Interestingly, research has revealed that the relationship between app use and physical activity level is mediated by the level of social support experienced [55]. As physical activity, even in acute doses, is known to enhance mental health (e.g., increase positive affect, decrease negative affect) [6], having a social component may be of integral importance to sustain physical activity in service of improving mental health.

### Limitations and future directions

We acknowledge several limitations of the current study. First, the nature of conducting this intervention online through Zoom excludes those who are unfamiliar with the platform and broadly, individuals who are not computer savvy. We also did not include a control group; future RCTs are warranted to show causal evidence that participating in an online dance lesson improves affective state and social connectedness. Second, our sample was made of primarily females (91.5%), perhaps because our methods of recruitment targeted more middle-high income, non-hispanic, normal weight female dancers. Additional recruitment methods will need to be intentionally incorporated in future research studies (e.g., recruiting from dance crews through flyers or other targeted advertisements). There was also a risk for selection bias in our recruitment because individuals with more positive experiences with dance may have been more willing to participate than those with more negative experiences. Though there was an equal sampling between urban and rural communities, future iterations should include more racially and ethnically diverse populations. As noted earlier, our target group consisted of healthy adult dancers without mental health issues; however, it is possible that online dance could be beneficial for clinical populations, such as those diagnosed with depression or anxiety, and it will be valuable to target these populations for future studies. Additionally, only three dance styles were represented based on the expertise of the instructors (ballet, jazz and contemporary/modern); we see potential for future expansion of the project into hip hop, tap, non-Western forms, or social dances, which could also diversify our group of participants.

Based on the current findings, we suggest some potential directions for future research. First, though this study was sufficiently powered for its cross-sectional nature, future studies should increase the sample size, perhaps including comparisons between sexes or differences seen across dance styles. Moreover, this pre-post study design with self-report answers gave insight on how participants felt immediately after a dance class. However, we anticipate that a long-term randomized control intervention of 4 weeks or longer with an appropriate control group (e.g., online discussion group, group movie watching, yoga, or other exercise) would help to demonstrate a significant effect of dance on psychological state and corroborate the findings of the present study.

Second, future work will need to investigate measures beyond self-report, such as neurocognitive assessments as well as the neural mechanisms underlying the beneficial effects of online dance on mental and social well-being through neuroimaging techniques such as functional magnetic resonance imaging. We also see potential in how this intervention could be applied to other forms of exercise or movement classes (e.g., yoga or other mindfulness practices), as well as its application in clinical populations, such as individuals with autism spectrum disorder, depression, anxiety, and posttraumatic stress disorder.

## Conclusions

The present study indicates that online dance is associated with acute increases in positive affect and self-esteem, decreases in negative affect and depressive symptoms, and enhancements in social and community connectedness for healthy adult dancers. Importantly, dance-induced affective state improvements predicted gains in social connection, demonstrating a body-mind-community connection. Future studies should utilize a randomized controlled design and increase sample size and population diversity in order to examine the utility of online dance to enhance mental and social health. Ultimately, we anticipate that online dance can be used as a tool to support mental wellness and social connection across diverse populations.

## Data Availability

All data is freely available upon request to the corresponding author.
